# Inefficiencies in the division of labour in human societies

**DOI:** 10.1098/rstb.2023.0278

**Published:** 2025-03-20

**Authors:** Claudia Diehl, Peter Preisendörfer

**Affiliations:** ^1^ Department of History and Sociology, University of Konstanz, Universitätsstrasse 10, Konstanz D-78457, Germany; ^2^ Institute of Sociology, University of Mainz, Jakob-Welder-Weg 12, Mainz D-55128, Germany

**Keywords:** division of labour, efficiency, social norms, power

## Abstract

The article reviews the long-standing debate on the division of labour in human societies from a sociological perspective. The division of labour is analysed as a secular trend towards increasing specialization on the one hand and as prevailing arrangements of specialization on the other. The dominant view in economics and other social sciences is that division of labour exists in human societies because it is efficient. We cast doubt on this view by discussing objections to the efficiency paradigm. We show that efficiency considerations, while important, are ultimately insufficient to explain both increasing specialization over time and prevailing arrangements of specialization in real life. As a broader framework, we briefly outline an explanatory triad of efficiency, norms and power. Social norms and power relations often complement unclear and ambiguous efficiency and performance criteria, but they can also conflict with principles of efficiency and rationality.

This article is part of the theme issue ‘Division of labour as key driver of social evolution’.

## 1. Introduction

The social sciences can look back on a long-standing debate on the social division of labour. This debate focuses on two closely related but analytically distinct issues: (i) the division of labour in terms of an increasing specialization of tasks over time; and (ii) the division of labour in terms of prevailing arrangements or patterns of specialization. The first issue, the secular trend of increasing specialization and its causes and consequences, has been most prominent among classic writers in the social sciences. More recent research, however, especially in the fields of organizational sociology and economics, has shifted attention to describing and explaining specific arrangements of the division of labour that can be observed in real life.

The purpose of this paper is to present a sociological perspective on these two strands of the division of labour debate. The particular point of view of sociology is a sceptical attitude towards a predominantly efficiency-oriented explanation of the division of labour. We begin by discussing what classic writers in sociology and economics have said about the promises and shortcomings of the secular trend towards an increasing specialization (§2). We show that considerations of performance and efficiency have indeed played an important role in their arguments. However, highly divided and specialized work is regularly associated with problems and disadvantages. We will review some of these problems, which may be interpreted as indications of ‘inefficiencies’. Yet, economists typically use a more specific notion of efficiency. Efficiency is an optimal use of scarce resources (labour, capital, etc.) to achieve intended outcomes. Applying this definition, we turn to the second strand, which deals with observable arrangements of specialization in real life (§3). In trying to evaluate these arrangements, it turns out that in many cases the criterion of efficiency is unclear and ambiguous, remains controversial and depends on the historical and social context. This leads to the question of alternative factors influencing the division of labour, especially social norms and power. As an example of the complexity of observable modes of specialization in human societies, we take a closer look at the division of housework between men and women. The article concludes with some final remarks (§4).

## 2. Classic writers: efficiency and disadvantages of increasing specialization

The crucial role of a more detailed and fine-grained division of labour in the evolution of human societies is a recurring and essential theme of classic writers in sociology and economics. By highlighting five of these writers in this section (Durkheim, Parsons, Luhmann, Smith and Weber), we will demonstrate that the division of labour is a fundamental organizational principle of human societies, that increasing specialization is often associated with higher performance and efficiency, but that high specialization also has a number of well known costs and drawbacks.

### (a) Specialization as secular trend: the Durkheim tradition

In his influential book *De la division du travail sociale* (*The Division of Labor in Society*), first published in 1893, Durkheim [1], one of the founding fathers of sociology, distinguishes two types of societies: simple and advanced. Simple societies have a limited degree of division of labour. They consist of segmented units (e.g. tribes, clans or villages) that share similar organizational principles. Individuals in these units typically perform a wide range of tasks necessary for the survival of the unit. They share common values, have a common worldview and have developed a common social conscience. These societies are held together by what Durkheim called ‘mechanical solidarity’.

Advanced societies, on the other hand, are characterized by a deeper and more far-reaching division of labour. Rather than consisting of a series of segmented but similar units, they are functionally differentiated, i.e. different units (e.g. occupational groups) focus on specific tasks while ignoring other tasks that are indispensable for everyday life. As a consequence, both individual actors and social groups are highly interdependent and structurally interwoven. They must rely on others to perform the tasks assigned to them. For advanced societies, Durkheim postulates ‘organic solidarity’ as the crucial mechanism of social cohesion. Organic solidarity is based on contractual arrangements and the recognition of interdependence, but its ultimate foundation—as with mechanical solidarity—is shared moral beliefs and social norms. Thus, Durkheim insists that advanced societies, too, rest on shared values, moral commitments, relationships of trust, and accepted social norms.

Building on Durkheim’s norm-focused sociology, Talcott Parsons, one of the leading figures in sociological theory in the 1950s and 1960s, continued the advance of social theory on the division of labour and social evolution [2,3]. According to Parsons, six ‘evolutionary universals’ are essential in the transition from less advanced to modern societies: the development of (i) a system of social stratification, (ii) patterns of cultural legitimation, (iii) bureaucratic organizations, (iv) money and markets, (v) a universalistic legal system, and (vi) democratic associations. All of these universals imply specialization and differentiation, lead to a higher level of societal complexity and contribute to an increase in the adaptive capacity of a society. Parsons [3] conceptualizes modern societies in terms of systems theory. The system of modern societies includes the evolutionary universals as structural components—components that fulfil the functional prerequisites of a social system.

Borrowing both from Parsons’ system theory and from Maturana and Varela's biological system theory , Niklas Luhmann [4,5], a renowned German sociologist in the Durkheim tradition, focuses on the feature of functional differentiation as a key characteristic of modern societies. Luhmann sees modern societies as a system of highly specialized subsystems (political, economic, legal, scientific, etc.), which are relatively independent of each other and maintain boundaries that limit the influence of other subsystems. The subsystems tend to operate ‘autopoietically’, i.e. they follow their own ‘binary codes’, have their own ‘internal logic’ and prefer internal communication. For example, while the code of the economic system is money, the code of the political system is power. The autopoiesis of the subsystems contributes to an enormous growth of their performance, but, at the same time, complicates and endangers the social integration of the whole society. According to Luhmann, functionally differentiated societies have particular difficulties in dealing with problems that cross-cut different subsystems.

### (b) Efficiency and rationalization: Adam Smith and Max Weber

While Durkheim, Parsons and Luhmann mainly described and portrayed the secular trend of specialization, Smith and Weber added efficiency and rationalization as factors more or less directly related to specialization.

Adam Smith, the founding father of modern economics, is still the most prominent reference for the position that increased specialization and efficiency are bound together. In book I, chapter 1 of his book *The wealth of nations*, first published in 1776, he gave the famous example of pin production [6, pp. 11−12]. Based on his own observations in a small pin factory and written reports from the French trade of pin makers, he compared the production of pins without and with division of labour. Without division of labour, he claimed that an experienced worker could not produce more than 20 pins a day. However, with division of labour, i.e. a pin production process divided into about 18 different operations assigned to specific workers, 10 workers were able to produce more than 48 000 pins per day. This means a daily output of 4800 pins per worker. Undoubtedly, 20 pins versus 4800 pins is an impressive difference.

Smith explicitly argues that the division of labour in an economy is not ‘the effect of any human wisdom, which foresees and intends that general opulence to which it gives occasion’, but an unanticipated consequence of the activities of individual actors following their ‘propensity to truck, barter, and exchange one thing for another’ [6, p. 18]. That is, the growing division of labour is a macro-level outcome, an emergent aggregate byproduct of the daily behaviour of individual actors who are trying to improve their quality of life. However, when we focus on the micro and meso levels, such as managers’ decisions about how to organize work in a firm, or household members’ routines about responsibilities for daily family tasks, Smith’s conviction is that actors are inventive and tend to follow their rational self-interest, and this most often leads them to organize a firm or a family as smoothly and productively as possible. Thus, we can interpret Smith to mean that the division of labour in his pin factory has its ultimate roots in intentional individual behaviour (of both managers and workers) that seeks efficiency in a learning process of trial and error.

In book I of *The wealth of nations*, Smith presents some arguments that the division of labour is good not only for the economic output but also for the workers involved in the production process. His main argument is that specialized tasks reduce the ‘mental work burden’. Workers do not have to shift their attention all the time, they have fewer distractions, and this requires less cognitive effort. With much more verve, however, Smith diagnoses in book V of *The wealth of nations* deleterious effects of a strong division of labour. It is ‘mentally stultifying’ for workers and ‘morally degenerating’. To prevent workers from becoming ‘stupid and ignorant’, he recommends, among other things, better education and more schooling for young working-class people, and more stimulating and challenging activities for workers outside the workplace.

The idea of efficiency also plays a crucial role in the work of Max Weber, one of the acknowledged founders of sociology and modern social theory. In his book *Wirtschaft und Gesellschaft* (*Economy and society*), first published in 1922, and in several of his other publications (e.g. his essays on the sociology of religion), he postulates a long-term evolution of Western societies towards increasing rationalization [7]. According to Weber, this process of rationalization can be observed in the sphere of world views and belief systems, in the sphere of social and political institutions and in the sphere of routines of the private life. In all three spheres, but especially in the sphere of institutions, Weber sees bureaucratic organizations as the main vehicle of social change towards rationality and efficiency.

He describes an ideal type of bureaucratic organization in modern societies with a set of essential characteristics: the organization consists of pre-defined positions; these positions have clearly defined rights and duties and are hierarchically structured; the holders of the positions follow given rules and act independently of their own interests and the special interests of those whom they serve; all processes and decisions are documented in written form; and the holders of the positions are specifically qualified for their jobs. These features serve as guidelines for bureaucratic organizations, although real-world organizations often fail to fulfil them. According to Weber, specialized organizations are ‘engines of efficiency’.

Like Smith, Weber had an ambivalent view of the division of labour and bureaucratic organizations. On the one hand, he admired the efficiency and superiority of bureaucratic organizations. During his research visit to the USA, for example, he was deeply impressed by the professional functioning of the American political parties, the labour unions and large corporations (e.g. the Chicago stockyards). The functioning of organizations follows the idea of legal authority, which is guided by general rules and professional behaviour. Compared with traditional authority and charismatic authority, legal authority has the advantages of accepted legitimacy and of predictability of outcomes. On the other hand, Weber was very concerned about the power, rigidity and impersonality of bureaucratic organizations. The implementation of general rules and organizational routines tends to ignore special circumstances, stifle innovation and creativity, and endorse the diffusion of responsibility. Weber famously summarized his concerns about bureaucracy, rationalization and the division of labour under the label of the ‘iron cage’, in which humanity is imprisoned.

### (c) Drawbacks of specialization

Following in the footsteps of Smith and Weber (and their many successors), we can safely conclude that efficiency and—more generally—considerations of performance and rationality are important and probably the essential correlates of increased specialization. This is confirmed by not only anecdotal evidence (à la Smith’s example of pin production) but also a broad stream of social science research. Nevertheless, it is important to recognize that the benefits of an increased division of labour do not come without costs. These costs and risks appear at different levels of society. We will briefly consider the micro level of individual workers and the macro level of the division of labour between nations.

At the level of the individual worker, a detailed division of labour (e.g. in the form of Taylorism and Fordism) contributes to ‘alienation’ from work, a concept originally introduced by Karl Marx (for a meta-analysis of alienation studies, see [8]). It is well known that highly specialized industrial working conditions threaten workers’ physical and mental health. Workers tend to respond to repetitive and boring tasks with a higher likelihood of quitting their job, higher rates of absenteeism, and various forms of dysfunctional behaviour (shirking, defective work, etc.). These negative reactions increase the production costs, and thus diminish the benefits of the division of labour. In the automobile industry, for example, high turnover and absenteeism rates among assembly line workers have been one of the reasons why the degree of division of labour has been reduced in the decades since the 1980s; workers’ jobs have become more enriching, i.e. more challenging and demanding, and now allow for higher skill levels [9].

Regarding the division of labour between nations, there has been a revival and a massive expansion of international economic cooperation since World War II, with a particular surge in the period from the mid-1990s to the global financial crisis of 2007/2008. This development, usually summarized under the label of ‘globalization’ or—for the latest boom period—‘hyperglobalization’ [10–12], has been associated with an intensive deepening of the international division of labour (in the form of migration of entire industries, just-in-time production, outsourcing, global supply chains, etc.). The basic proposition of advocates of the globalization process is that the international division of labour ultimately benefits all participating countries, because nations can and will specialize in those activities for which they have comparative advantages of production. Since the global financial crisis, however, critics of globalization have become more vocal, and even its proponents have begun to see significant risks. The worldwide economic growth fuelled by globalization is increasingly endangering the environment (loss of biodiversity, climate change, etc.); the benefits of globalization are very unevenly distributed; the mutual dependencies create the risk of opportunism and exploitation; and complex supply chains lack stability and robustness.

## 3. Modern organization studies: inefficiencies of observed arrangements of specialization

The classic research agenda, devoted to increasing specialization over time, has been fruitful mainly in analysing specialization at the macro-societal level (within and between nations). For more detailed and fine-grained analyses of the division of labour at the micro and meso levels (in settings such as firms or families), however, the focus on increasing specialization is too narrow, and the diagnosis is often empirically wrong. Despite a secular trend, there is no ‘iron law’ of increasing specialization. The strength of the division of labour may decline (see our example of the automobile industry above), and technological innovations may introduce new production processes that cannot be easily described along a line of more or less specialization.

In light of this, recent research on the division of labour, which is predominantly rooted in organizational economics and sociology and focuses on organizations as epitomes of specialized work, has shifted attention to describing and explaining specific arrangements or patterns of specialization. In real life, we can observe prevailing arrangements of the division of labour in firms and other organizations that have evolved over time. The question is how and why these particular arrangements have become dominant. Much like in the debate on increasing specialization, the most frequent answer is that efficiency is the ultimate driver—efficiency in the sense of an optimal use of resources to achieve a given outcome. It is argued that in a learning process of ‘cultural evolution’ [13–15], new arrangements of the division of labour are discovered and explored, and the efficient ones will eventually spread and survive. But does this efficiency paradigm, which is particularly prominent in economics, hold up, and what are the objections to it?

### (a) Beyond efficiency

Within economics, the most influential opponent of the efficiency paradigm is the transaction cost theory of Williamson [16,17]. Regarding the division of labour between organizations, for example, Williamson begins with the observation that organizations often do not practise the most cost-effective form of division of labour, but rather use more complex arrangements of organizational cooperation. Transaction cost theory assumes that an organization always has to decide whether to produce the goods and services needed for its own production itself or to buy them from outside, most often from another organization (make or buy decision). A buy decision implies the creation of a dependency relation, which is associated with the risk of opportunistic behaviour on the part of the transaction partner. When an organization relies on a single supplier for a necessary input, a ‘lock-in effect’ is the result, making it difficult to exit the transaction without incurring high costs. To avoid a lock-in, organizations typically use more than one supplier for a necessary input, even though short-term cost calculations would recommend choosing only the one supplier that currently offers the best deal. In addition to this strategy of supplier diversification, organizations use a wide range of sophisticated strategies to regulate and stabilize their relationships with other organizations—strategies that at first glance appear economically ill-advised. These strategies include the gradual expansion of transactions, unwritten agreements, personal trust relationships and reputation networks (for more on the role of trust and networks, see [18]).

Turning to the sociological perspective that guides the argument in this article, in particular the theoretical approach of new institutionalism (for initial contributions, see Meyer & Rowan [19] and DiMaggio & Powell [20]) has set out a research programme that denies the dominance of economic efficiency in the real world of organizations and other spheres of social life. For Meyer & Rowan, the ideas of efficiency and rationality of organizations are social ‘myths’ that are more or less successfully cultivated and celebrated by organizations and their agents. In terms of a ‘new’ perspective, proponents of the new institutionalism agree on the agenda of looking for ‘factors beyond efficiency’ that influence social practices and observable arrangements of the division of labour.

The critique of the efficiency paradigm can begin with a debate on the concept and definition of efficiency. Obviously, efficiency is more than just short-term financial profitability—a definition often proclaimed in management circles. In addition to monetary aspects, organizational routines and governance structures (design of working conditions, customer treatment, firm networks, etc.) have social and political impacts that should also be taken into account for a proper evaluation. Although economists have developed various techniques of cost–benefit analysis that attempt to incorporate non-monetary impacts by monetarizing them [21], in many cases this approach depends on shaky assumptions and problematic measurement devices. For example, it is difficult to monetarize the low job satisfaction of assembly line workers, the feeling of powerlessness of customers in their transactions with large corporations, or the loss of reputation of a company that makes profits by exploiting lock-in effects.

Related to this, short-term efficiency often diverges from long-term efficiency. Closing departments and firing workers during a recession period immediately reduces a company’s costs. However, it may be an unwise decision, because the demand may increase in the next period and experienced workers may then be unwilling to return. Short-term profitability (efficiency) is easier to calculate than long-term profitability (efficiency) because the latter pertains to the uncertain future. This is probably the reason why we observe in the real world a preference for short-term profitability (efficiency). An understanding of efficiency that includes non-monetary outcomes and refers to a longer period of time implies a decision criterion that is more vague and controversial than that based on limited short-term profitability.

Other real-world examples suggest that seemingly insufficient and inefficient arrangements of division of labour persist because of ‘path-dependency’ [22,23]. Historical decisions and developments have often produced a *status quo* that is relatively stable and difficult and costly to change, even though experts agree that there are better, more efficient alternatives. The persistence of ‘deviant outcomes’ can be the result of vested interests and economic or political power, but also elementary economic reasons (high switching costs, loss of skills, transaction costs, etc.) can be responsible for the persistence of inefficient second-best solutions. There are many examples of path dependencies in the fields of technological devices and social institutions and governance structures.

Regarding the role of the future, it is a commonplace that the future is uncertain and difficult to predict. Although researchers attempt to capture this uncertainty by discounting future costs and benefits, the discount rates require assumptions that may turn out to be incorrect. In particular, it is difficult to predict the success of technological and organizational innovations that may accelerate or reshape the social division of labour [24]. Most technological innovations and new organizational forms (if they are more than incremental) are initially inefficient owing to a lack of experience, knowledge, customer acceptance and general legitimacy [25, chs 10 and 11]. Early adopters are often techno-enthusiasts, visionary entrepreneurs or management gurus who are not put off by ‘start-up frictions’. It is an empirical fact that most innovations ultimately fail; in the words of Levitt & March [26, p. 334]: ‘it seems axiomatic that most new ideas are bad ones’. Moreover, many innovations find their way into applications and markets that the original innovators did not anticipate. Applications of artificial intelligence may serve as a recent example.

The time-dependence of efficiency is a variant of the broader insight that the context affects efficiency. A sophisticated technology or institutional arrangement that is helpful and appropriate in one context may be inappropriate in another context. Numerous examples of this can be found in projects that have attempted to transfer successful technologies or institutions from one country (e.g. a wealthy Western country) to other countries (e.g. less developed countries). Ultimately, this means that efficiency is not an attribute of a technological or organizational arrangement but the result of the arrangement’s fit with a given context.

The uncertainty of the future and the dependence on context make it possible to view efficiency as a matter of controversy and debate. The fate of a technology, an institution or an organizational form is often determined not by the objective but by the subjective, i.e. perceived efficiency. Proponents of the new institutionalism are convinced that strategies of impression management and self-presentation play an important role in organizational and other settings. They diagnose a frequent discrepancy between ‘talk and action’ [27]. Managers of economic, political and social organizations are trained to produce positive and encouraging news about their organizations, and perceived performance is often more important than actual performance. That one’s organization uses its resources efficiently is one of the standard ‘narratives’ and ‘myths’ of organizational life [19]. The example of ‘woke capitalism’, i.e. the actual or proclaimed commitment of firms—including those with a history of tax evasion and worker exploitation—to act in accordance with societal debates about anti-racism or anti-sexism, can be seen as such a deviation from the division of labour between societal subsystems [28]. After all, following moral criteria could be considered a waste of time and money. However, such an investment in impression management may yield payoffs in the long run by pleasing a certain clientele.

### (b) Norms and power as influence factors

All of these objections to the efficiency criterion raise the question of whether there are alternative criteria that can better explain the evolution of organizational practices and specific arrangements of division of labour. Since this is not the main thrust of our article, we will only briefly comment on it in the following.

Advocates of the new institutionalism propose ‘legitimacy’ as an alternative. For their success and survival, organizations and (new) organizational practices depend on the support and acceptance of their external environment. Legitimacy is a prerequisite for ensuring a continuous inflow of resources from direct interaction partners (suppliers and customers) and from the broader society. New institutionalists argue that organizations often adopt costly and inefficient routines and practices because of legitimacy considerations. The quest for legitimacy creates a tendency towards ‘isomorphism’, i.e. a structural similarity between organizations and their environment [19,20]. Structural similarity mainly means alignment with prevailing social and cultural norms in a society. Thus, we can conclude that—in the tradition of Durkheim—adherents of new institutionalism focus on the role of social norms rather than on considerations of efficiency as drivers of the selection of organizational practices.

In line with other authors [29], we believe that economic efficiency on the one hand and compliance with social norms on the other are not necessarily contradictory but often corresponding or complementary criteria. For example, if a for-profit company seeks to build up a high reputation by complying with social norms (legitimacy), this may also help to increase financial profit (efficiency). And *vice versa*, financial success often helps improve reputation (e.g. because a company can initiate activities that signal corporate social responsibility). In short, in the real world, often both criteria matter.

Finally, in addition to economic efficiency and norm conformity, we should not forget a third elementary factor that is responsible for observable arrangements of division of labour: power and coercion. DiMaggio & Powell [20] introduce this factor with their concept of ‘coercive isomorphism’. This means that organizations conform to social norms because the state compels them to obey laws and other public regulations (e.g. minimum wages, safety regulations, environmental standards, etc.). But power and coercion go much further than government regulation. In general, a position of power makes it possible to establish and maintain a personal or organizational relationship that unilaterally serves the interests of the dominant actor. Whether the outcome of this relationship is efficient or conforms to social norms is not a primary but at best a secondary factor. In the history of human societies, power-based social relations and coercive organizational arrangements are common and pervasive. The most influential tradition in sociology that centres on power as the primary driver of social organization is Marxist theory (for an introduction with special attention to the division of labour, see [30]).

An example that has received much scholarly attention in this context is ‘slave labour’ (for an overview, see [31]). It is an empirical fact that slavery, which by definition is based on coercion, has been widespread throughout human history and still exists in some parts of the world. Many historians (but with prominent exceptions, see [32]) argue that slavery was abolished primarily because of its economic inefficiency. In addition to its violation of basic human rights and its immorality, i.e. problems of legitimacy and of conformity with contemporary norms, slave labour has several economic shortcomings: it requires intensive monitoring and control of the workers, it destroys intrinsic motivation to work, and it is ultimately a waste of human resources. Historical research on slavery reflects and reiterates the three influence factors on social and organizational arrangements that we have tried to elaborate: economic efficiency, social and cultural norms in a society, and power and coercion.

### (c) Example: division of household tasks between men and women

To illustrate how difficult it is to explain real-life arrangements of the division of labour in terms of efficiency, norms and power, we will finally take a closer look at the gendered division of labour within households. This strand of research analyses how housework is divided among cohabiting couples. We have chosen the household example to make clear that the debate about particular modes of division of labour is not only limited to formal organizations but also concerns more informal and personalized social groups.

Research in many countries shows that women do much more housework than men. For example, based on data from the World Values Survey for 80 countries, a recent UNDP report [33] found that, on average, women spend about twice as much time as men on domestic chores and care work. When both the woman and the man in a family have a paid full-time job, the total time per week spent on housework and childcare decreases (partly owing to outsourcing of tasks), but women still have a significantly higher share. Looking at different types of household tasks provides further insights into the picture of gender inequality. Taking the US situation as an instance, table 1 shows the roles of men and women for a set of 12 household tasks, based on a Gallup survey.

**Table 1.** Roles of men and women in US households. The data pertain to heterosexual couples who are married or living together. Respondents with ‘missing values’ and ‘does not apply’ are excluded. Survey question: 'Who is more likely to do each of the following in your household?' Source: Gallup Survey 2019. Table adapted from [34].

	% woman more likely	% man more likely	% both equally
make decisions about furniture and decorations	63	4	33
laundry	59	13	28
clean the house	53	9	38
prepare meals	51	17	32
care for children on a daily basis	51	7	42
grocery shopping	45	18	37
wash dishes	43	20	37
pay bills	37	34	29
plan family activities	37	10	53
make decisions about savings or investments	18	31	51
keep the car in good condition	12	70	18
yardwork	11	66	23

The first seven tasks in the table, ‘make decisions about furniture and decorations’, ‘laundry’, ‘clean the house’, ‘prepare meals’, ‘care for children on daily basis’, ‘grocery shopping’ and ‘wash dishes’, are clearly domains of women. The next two tasks, ‘pay bills’ and ‘plan family activities’, are relatively equally distributed between men and women. And the three remaining tasks, ‘make decisions about savings and investments’, ‘keep the car in good condition’ and ‘yardwork’, are men’s domains.

In trying to explain these arrangements of division of labour, it is plausible to assume that efficiency factors are at work. Probably the most prominent ‘efficiency theory’ regarding the division of labour within households can be found in Gary Becker’s book *A treatise on the family*, first published in 1981 [35, ch. 2]. Unlike other economists, who focus on the family as a consumption unit, Becker conceptualizes the family as a production unit. The ‘family firm’ attempts to efficiently transform ‘inputs’ (the time and other endowments of the household members) into valued ‘outputs’ (goods and commodities). To maximize the family’s utility, the household members allocate their time to different tasks inside and outside the household according to the principle of comparative advantage. This means that the person who can earn more money in the labour market should focus on paid work. Taking into account their limited time budget, family members should specialize in those tasks within the household where they are relatively better, i.e. relatively more efficient, than their partner. In light of the principle of comparative advantage, even small initial differences in skills and experience between partners usually lead to a complete division of labour.

Thus, for proponents of Becker’s theory, an unequal distribution of household tasks between men and women does not come as a surprise. On the contrary, the theory has trouble explaining why there is so much egalitarian task sharing within households. In table 1, the percentages of ‘both do it equally’ range from 18 to 53%. It is very unlikely that the constellation of no comparative advantage between men and women is so common for so many tasks. We should keep in mind, however, that Becker formulated a theory—and a theory can be wrong (for only weak empirical support of Becker’s comparative advantage explanation, see [36]).

While equal division of housework is widespread enough to cast doubt on Becker’s efficiency theory, norm-oriented explanations of the division of labour in households seem to have problems with too little equal division. The normative principle of an egalitarian partnership, which is prevalent in Western countries today, raises the expectation that men and women participate equally in housework and child care, and that there is no deep division of labour. The ‘both do it equally’ percentages in table 1 can serve as evidence that there is at least some effort to achieve a balanced partnership. However, an egalitarian partnership is difficult to reconcile with the fact that women are much more likely than men to be responsible for doing the laundry, cleaning the house and preparing meals, and—*vice versa*—that men are much more likely to have the responsibility for savings and investments, the car and the garden.

The differences we observe suggest that—in addition to efficiency factors and in competition with egalitarian norms—traditional family and gender role models influence the division of labour. Regarding the total amount of housework, the UNDP report already mentioned above [33] shows that the housework done by women increases with the value of a *gender social norms index* (GSNI), which summarizes the bias against gender equality in a country, as measured in the World Values Survey. In countries in the upper quartile, Q4, of the GSNI, i.e. in countries with low support for gender equality, the female-to-male ratio of time spent on unpaid domestic chores and care work is more than six times higher than in those countries that fall in the lowest quartile, Q1. High levels of religiosity have also been shown to go along with more traditional division of labour arrangements between couples [37].

More fine-grained sociological studies reveal that the latent tension between modern and traditional family and gender roles can have ‘strange effects’. One such effect is claimed by the gender deviance neutralization hypothesis [38,39]. This hypothesis predicts that women who earn more money in their jobs than their male partners, as compared with women who earn less, do more housework to compensate for having violated the ‘male breadwinner’ norm. Or, more generally, when women outperform their male partners in the labour market, they attempt ‘doing gender’ in the household to neutralize the deviation from the traditional norm. This means that housework serves as a symbolic enactment of gender relations. Although some studies found empirical support for this hypothesis, the overall picture is one of mixed evidence (for a brief review, see [40]).

Those who argue that power (the third factor in the triad) influences the division of labour in households theoretically disagree with the neutralization hypothesis. To bring power in, they usually follow models of bargaining power in the family, preferably the relative resource-bargaining perspective (introduced by Blood & Wolfe [41]). Blood & Wolfe propose that the amount of housework and the division of family tasks are determined by the distribution of relative resources between the partners. The more resources (education, occupational status, income from paid work, etc.) a person has relative to his/her partner, the more likely he/she will negotiate housework away. This is a more general perspective than the idea in Becker’s theory that the bargaining power in the marriage market explains the distribution of the jointly produced outcomes in the family. Indeed, there are many empirical studies that tend to confirm the predictions of bargaining power models, but the ‘power effects’ are mostly moderate in strength (for a brief review and a useful empirical study, see [42]).

Taken together, we conclude: how much housework men and women do and how they divide up the various tasks is influenced to some extent by considerations of efficiency and by the bargaining power of the partners in their relationship. In addition, an explanation of household routines definitely cannot do without reference to social norms, in this case norms that represent modern and traditional family and gender role models.

## 4. Concluding remarks

Division of labour is found in a number of species. In human society, however, it is far-reaching and all-pervasive. At the macro level, it seems to be a general rule that the depth of the division of labour increases with the level of modernization in human societies. Recent research, however, has shifted attention away from the historical trend towards increasing specialization. It focuses primarily on the meso level of organizations and analyses prevailing arrangements of the division of labour as they are observed in real life. In explaining both increasing specialization and specific arrangements of specialization, the dominant view in economics is that efficiency considerations are the essential driving force.

Taking a sociological perspective, we have tried to show in this article that efficiency considerations are important but ultimately insufficient for understanding the diverse manifestations of the division of labour in real life. There are serious objections to the efficiency paradigm, discussed in organizational sociology under the heading ‘beyond efficiency’. As a broader framework, we have briefly outlined the explanatory triad of efficiency, norms and power. Historically, Smith, Durkheim and Marx paved the way for this triad. Social norms and power relations can complement unclear and ambiguous efficiency and performance criteria, but they can also contradict efficiency guidelines. Our example of the division of housework between men and women highlighted the role of social norms. In other contexts, as the example of slave labour shows, power and coercion are obviously more dominant.

Since organizations are the crucial context in which the division of labour unfolds in modern societies, we are convinced that the study of organizations is the research field that deserves pivotal attention in the debate on the division of labour. With the transaction cost theory and the approach of new institutionalism, the field of modern organization studies offers two theoretical perspectives that can be fruitful and inspiring for research on both the strength and the modes of division of labour. A particular advantage of focusing on the meso level of organizations is that research results can also help to address practical problems. We live in a society of organizations, and their design directly affects the living conditions of many people. Organizations are products of human action; their division of labour and, related to this, their organizational routines and practices should serve human needs and improve our quality of life.

## Data Availability

This article has no additional data.
